# PD-L1: Biological mechanism, function, and immunotherapy in gastric cancer

**DOI:** 10.3389/fimmu.2022.1060497

**Published:** 2022-11-24

**Authors:** Yingzi Zhang, Yan Yang, Yiran Chen, Wu Lin, Xiangliu Chen, Jin Liu, Yingying Huang, Haiyong Wang, Lisong Teng

**Affiliations:** Department of Surgical Oncology, The First Affiliated Hospital, School of Medicine, Zhejiang University, Hangzhou, China

**Keywords:** gastric cancer, programmed death-ligand 1, immune checkpoint inhibitor (ICI), clinical trials, tumor microenvironment

## Abstract

Gastric cancer (GC) is one of the main causes of cancer incidence rate and mortality worldwide. As the main breakthrough direction, the application of immune checkpoint inhibitors makes patients with GC have better prognosis, where PD-L1/PD-1 inhibitors in immunotherapy have good anti-tumor immune efficacy. Further understanding of the regulatory mechanism of PD-L1 in GC may bring substantial progress to the immunotherapy. In this review, we provide information on the endogenous and exogenous regulatory mechanisms of PD-L1 and its biological functions combined with current clinical trials of PD-L1/PD-1 inhibitors in GC. The malignant biological phenotypes caused by PD-L1 and the corresponding clinical combined treatment scheme have been reported. Identifying the biomarkers of the potential efficacy of immunotherapy and specifying the clinical immunotherapy scheme in combination with molecular characteristics of patients may maximize clinical benefits and better prognosis.

## 1 Introduction

Gastric cancer (GC) is one of the five most common cancers in the world and the fourth most common cause of cancer death, is responsible for over one million new cases in 2020 and an estimated 769,000 deaths (1). Helicobacter pylori infection, special age and high salt diet may induce the occurrence of GC (2). The Cancer Genome Atlas (TCGA) research network has published the latest genetic classification of gastric cancer, including Epstein Barr virus positive (EBV+), microsatellite install (MSI), genomically stable, and chromosomal unstable (CIN) (3). Surgical or endoscopic resection is still a mandatory backbone in treatment with curative intent. Continuous chemotherapy is used in the treatment of advanced GC, and platinum and fluoropyrimidine combined chemotherapy is the first-line treatment. Clinically, patients with GC, especially advanced GC, chemotherapy is a routine treatment, commonly include fluorouracil (5-FU)/capecitabine, paclitaxel (paclitaxel or docetaxel) and platinum, or a combination of these chemotherapeutic drugs (4). Targeted therapies for GC have been reported as follows: trastuzumab for the treatment of human epidermal growth factor (HER2) positive patients, Ramoximab for specific targeted angiogenesis, and anti-PD-L1(programmed cell death-Ligand 1)/PD-1(programmed death-1) inhibitors for patients with advanced GC, including nivolumab or pembrolizumab (5). Tumor immunotherapy is a sort of new tumor treatment which has developed rapidly compared with traditional treatment such as surgery, radiotherapy and chemotherapy and has great clinical application prospects. Immune checkpoint blockade is now established as a sort of combination treatment for chemorefractory gastric cancer (cancer which has progressed after two or more lines of chemotherapy) (6). However, such monitoring is not always perfect, tumor cells can escape immune regulation through a variety of ways under certain conditions. Compared with traditional treatment, tumor immunotherapy has multiple advantages of strong specificity and less side effects, which could provide a prospective view for treatment of solid tumors (7). At present, cancer immunotherapies include immune checkpoint inhibitor therapy, adoptive cell immunotherapy(ACT), cancer vaccine and some emerging immunotherapy (including LAG3、TIM3、VISTA and B7-H3). This article mainly discusses the regulation and biological consequences of intracellular PD-L1 expression level and the clinical application of PD-L1/PD-1 inhibitors in immunosuppressive checkpoints in GC.

In gastric adenocarcinoma (8, 9), there are tumor-infiltrating lymphocytes (TIL) in the tumor area or the surrounding matrix. The effectiveness of antitumor immune response may bring a better prognosis for patients with this type of GC. After curative surgery, patients with high TIL tumors had better disease-free survival (DFS) and overall survival (OS) than patients with low TIL tumors (8). Inhibiting the expression of PD-L1 on tumor cells can enhance immune surveillance and reduce the function of PD-L1 derived immune checkpoint (10). Agents against PD-L1/PD-1 have shown significant clinical significance in non-small-cell-lung-cancer(NSCLC) (11), melanoma (12) and GC (5) amongst others. Immune checkpoint receptor PD-1 is a co-inhibitory molecule, which physiologically expresses on the cell surface of immune cells, such as T and B lymphocytes or myeloid cells, and provides signal immune system activity to terminate immunity. PD-1 receptor has two natural ligands, PD-L1 (B7-H1) and PD-L2 (B7-DC). It can be expressed not only in immune cells, but also in tumor cells, and represents a potential mechanism of immune surveillance escape (13). In general, PD-L1/PD-1 axis is considered as a significant factor of poor prognosis across different types of GC patients. In this article we aim to describe the biological mechanism and function of PD-L1 regulation and clinical application of immune checkpoint inhibition as a therapeutic strategy in GC, and to discuss the progress and perspective on immune checkpoint inhibitors (ICIs).

## 2 Regulatory mechanism of PD-L1 in GC

**Figure 1:**
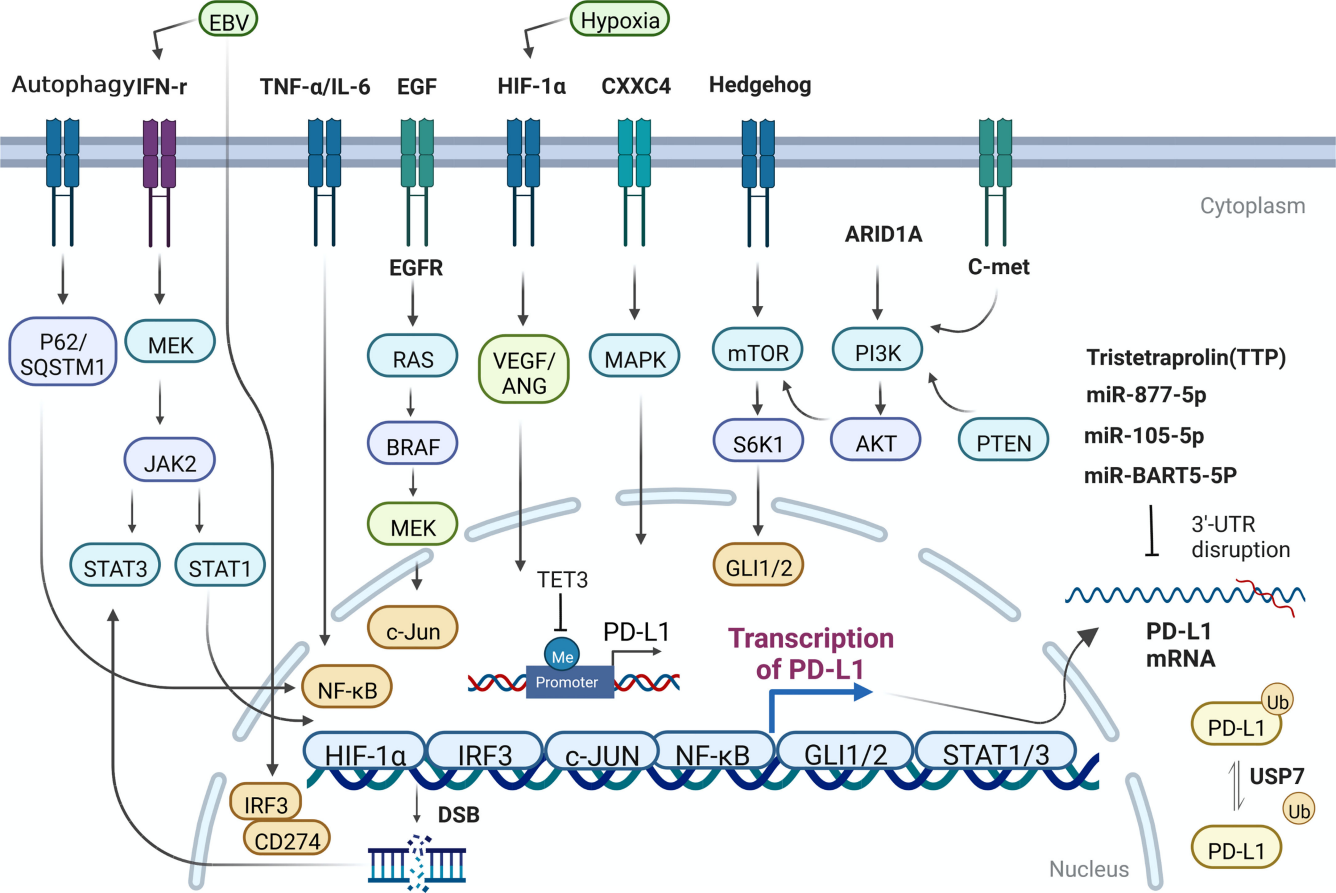
The regulatory mechanism of PD-L1 expression in GC cells. Me, methylation; Ub, Ubiquitination.

The regulatory mechanism of PD-L1 in GC is mainly summarized in **Figure 1**.

### 2.1 Regulation of PD-L1 expression

#### 2.1.1 Transcriptional regulation

The expression of PD-L1 can be simply induced by the change of gene copy number and chromosome rearrangement in the chromosome region (9p24.1) of PD-L1 and PD-L2 loci (14, 15). Such events mainly occur in hematological malignancies. Nevertheless, the destruction or mutation of the 3’ untranslated region of PD-L1 can directly affect its expression level in GC (16, 17). Some oncogenes have been identified, such as MYC, which regulates the anti-tumor immune response through CD47 and PD-L1, and ALK, which affect the expression of PD-L1 through STAT3 pathway. Concurrently, other molecules, including HIF1/2 α, hosting hypoxia-mediated escape from adaptive immunity, NF-κB, MAPK, mediating resistance to BRAF inhibition and upregulating PD-L1 expression, PTEN/PI3K, LKB1, loss of which could lead to elevated PD-L1 expression (18, 19). EGFR, whose activation closely relate to expression of PD-L1, contributes to immune escape in various tumors. PD-L1 has been reported to bind to EGFR and activate EGFR to promote the progression of GC (20). Expression level of PD-L1 was significantly inhibited when EGFR/HER2 signaling pathway was blocked in GC. Hypoxia-HIF-1α-VEGF/ANG signaling also weighs heavily in advanced GC (21). Overexpression of CXXC4 in GC leads to down-regulation of MAPK signaling pathway (22). For patients with MSI-H GC, who own better prognosis among diverse molecular types of GC patients, the expression of PD-L1 in ARID1A-deficient tumors was significantly higher (23). Hedgehog signal induces PD-L1 upregulation and tumor cell proliferation in GC (24). Nor is this all, the transcription effector Gli of hedgehog signal also can induce the expression of PD-L1 in GC through mTOR pathway (25).

#### 2.1.2 Regulation by post-transcriptional modification

The expression of PD-L1 in GC can also be regulated by miRNA and non-coding long chain RNA. For example, Mir-bart5-5p is expressed in EBV+ GC cells and specimens and promotes the expression of PD-L1 and the occurrence of GC subsequently through PIAS3/pSTAT3 pathway (26). Mir-105-5p, regulates PD-L1 expression and tumor immunogenicity in GC (27). LncRNA PROX1-AS1 promotes GC progression through mir-877-5p/PD-L1 axis (28). Tristetraprolin (TTP), a RNA-binding protein, binds to adenosine-uridine AU-rich elements in the 3’-untranslated region of messenger RNAs and facilitates rapid degradation of the target mRNAs. High TTP expression downregulates PD-L1 and proceed to affect GC cell survival and apoptosis, increasing peripheral blood mononuclear lymphocyte (PBML)-mediated cytotoxicity and slowing down tumor progression (29). One research in 2021 showed that PD-L1 was significantly hypermethylated during gastric carcinogenesis, and the DNA methylation of PD-L1 was negatively correlated with the expression of PD-L1 in GC samples (30). Meanwhile, some studies have also found that mir-105-5p is controlled by DNA methylation of Gabra3 promoter in GC, affecting the expression of PD-L1 and the progression of GC (27). In another study, after treating GC cells with compound oleanolic acid (OA), the expression specificity of DNA demethylase TET3 decreased and expression of downstream interleukin-1β (IL-1 β) was downregulated, so is PD-L1. In this process, DNA demethylase TET3, as an important mechanistic molecule, can largely restore the effect of OA on PD-L1 (31). Upregulation of MHC class I and MHC class II by DNA methyltransferases inhibitors (DNMTi) has appeared in many cancers (32). It is clearly reported that in gastric cancer, the association between PD-L1 positivity and histone N-methyltransferase 2 (KMT2) family member mutations remained significant in the proficient-MMR and microsatellite stable subgroup (33).

#### 2.1.3 Regulation by ubiquitination

Ubiquitination ligases can greatly affect tumorigenesis in different ways. For example, it can regulate the stability of oncogenes and tumor suppressor genes. Ubiquitination labeled proteins are transported to proteasome or lysosome for degradation. Ubiquitin specific peptidase (USP7) is a member of ubiquitination related protein family, which can promote the stability of various proteins. Wang, Z et al. demonstrated that USP7 was highly expressed in GC, and poorly differentiated patients with high USP7 expression has worse overall survival. USP7 can directly interact with and de-ubiquitinate PD-L1, and then enhance the immune escape level in tumor immunity and promote the progression of GC (34). Ubiquitination is an important mechanism for PD-L1 to maintain protein stability in cancers.

### 2.2 Extracellular microenvironment events

**Figure 2:**
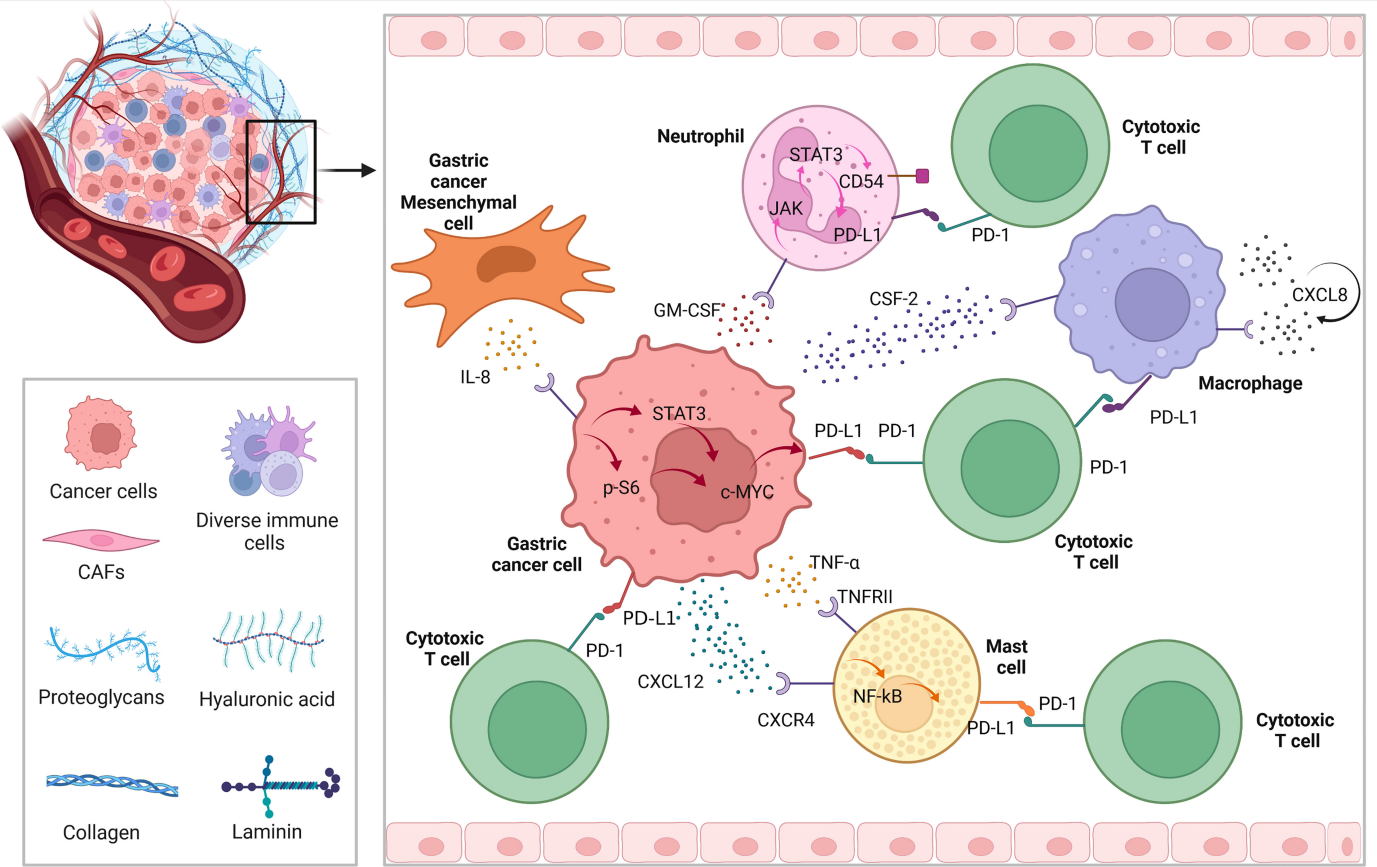
The regulatory mechanism of PD-L1 expression among tumor microenvironment in GC.

As we know, except for tumor cells, PD-L1 is highly expressed on the surface of tumor infiltrating immune cell, such as neutrophils, macrophages, and mast cells. Since GC is a solid tumor with relatively weak immunogenicity, we found that there are many studies related to immunotherapy of GC target the immune cells infiltrating around the tumor. **Figure 2** briefly describes the expression of PD-L1 on immune cells in the microenvironment of GC.

In GC, the expression of PD-L1 of neutrophils can be induced by tumor derived granulocyte-macrophage colony-stimulating factor (GM-CSF), which can be activated through Janus kinase (JAK) - signal transducer and activator of transcription 3 (STAT3) pathway, to a large extent, inhibiting tumor immunity and promote progression of GC (35). Similarly, while chemokine colony stimulating factor 2 (CSF-2) secreted by GC cells, macrophages are stimulated to secrete lots of chemokine CXCL8. In this autocrine form, macrophages continue to receive positive feedback, resulting PD-L1 highly expresses on the cell surface, to play the above tumor immunosuppression (36). In the tumor microenvironment, in some degree cytokines and chemokines are responsible for recruiting immune cells. Among them, GC cells can recruit mast cells through CXCR4-CXCL12 receptor-ligand binding. Then, under the condition that GC cells secrete a large amount of tumor necrosis factor a (TNF-a), while mast cells express TNF-a II ligand to receive such signals, and highly express PD-L1 through NF-kB pathway, to further promote the malignant progression of GC by cascade (37).

Induction and activation of JAK2/STAT3 signal by interferon γ (IFN- γ) can promote the upregulation of PD-L1 expression in GC and various kinds of solid tumors (38–40). In GC, in addition to the EGFR mentioned above, there are c-Met, mTOR, PI3K-Akt, JAK2/STAT3 and NF KB signaling pathways or key molecules (41, 42) that can make a difference to PD-L1. When it comes to STAT signal related genes, some researchers found that some DNA double strand breaks can activate STAT signal pathway and enhance the expression of PD-L1 (43). Besides, cytokine TNF- α and IL-6 also activate NF-KB and STAT3 signaling pathways in regulating the expression of PD-L1 (44). Other inflammatory cytokines, such as toll like receptor 3(TLR-3), transforming growth factor β (TGF-β), IFN- α/β and IL-4/6/17/27 have been shown to enhance the expression of PD-L1 mRNA on tumor cells or tumor associated stromal cells. In tissue samples from patients with GC, the expression of PD-L1 on tumor cell membrane was positively correlated with the presence of CD8 positive T cells and IFN- γ in matrix (45, 46). Overexpressing PD-L1 GC cell could recruit and enrich myeloid-Derived Suppressor Cells (MDSCs) by releasing lots of cytokines and promote the occurrence of gastric tumors by inhibiting tumor infiltrating CD8 + T cells (47). There are reports in the document that IFN- γ could restrain autophagy in the mechanism of promoting PD-L1 gene transcription (38, 40). Lately, Wang et al. found that autophagy, one specific mode of cell death, can directly down regulate PD-L1 through p62/SQSTM1-NF-κB pathway in GC (48).

### 2.3 Exogenous and special events

#### 2.3.1 Drug induced changes in the expression level of PD-L1

Gastrointestinal cancer cells can up-regulate PD-L1 under the condition of 5-FU treatment. The improvement of prognosis of patients with gastrointestinal adenocarcinoma has been shown in various RCTs of the combination of 5-FU and PD-1/PD-L1 inhibitors (49). In another study, exogenous PD-L1 treatment could also significantly enhance the apoptosis of Jurkat T cells and inhibit T cell activation in PBMC. After combining nivolumab, a PD-L1 inhibitor, the immunosuppression of T cells was significantly alleviated (50). Vivien Koh et al. found that rapamycin blocked the transcriptional regulation of PD-L1 by Gli1 and Gli2 signals (25).

#### 2.3.2 Abnormal expression of PD-L1 in GC specific cells

There are many different typing ways for GC cells, including molecular typing and histologic typing, such as EBV positive and MSI high cells. In addition, there are some special ones, such as GC cell lines with CSC characteristics (NCC-S1M), GC mesenchymal stem cell (GCMSC) and so on. NCC-S1M is the only widely recognized syngeneic GC transplantational model cell line. EBV+ GC cells not only promoted the amplification of *CD274* gene (encoding PD-L1) by activating IRF3/CD274 axis, but also caused the up-regulation of PD-L1 constitutive expression (51). EBV infection stimulated elevated IFN- γ in GC, inducing the enhanced adaptive expression of PD-L1 (51–53). In another molecular type of GC, the expression of PD-L1 was also up-regulated, in which patients have the better clinical prognosis (54–58). For instance, the potential mechanism of high expression of PD-L1 in MSI-high GC was reported by Fang et al., which may be related to secretion of lots of new antigen in GC cells, therefore T lymphocytes infiltrate and secrete a large amount of IFN- γ, leading to GC progression (59). It should be noted that CD274 is highly expressed in NCC-S1M cells compared with normal gastric tissues. Treatment with PD-1 inhibitor can effectively retard the tumorigenesis of NCC-S1M cells (60). Mesenchymal stem cells get many biological functions, including secreting various chemokines, promoting angiogenesis, maintaining cell stemness and cellular immunity. GCMSC can secrete IL-8 and promote the high expression of PD-L1 in gastric tumor cells *via* classic STAT3/mTOR-c-MYC pathway (61). According to a recent report that GCMSC can further enhance the CSC-like characteristics of GC cells through PD-L1 associated with CTCF, to promote the invasion and drug resistance of GC cells (62).

## 3 Biological consequences of PD-L1 expression in GC

### 3.1 PD-L1 and EMT

The increased expression of PD-L1 in GC is related to the epithelial mesenchymal transformation phenotype (EMT), which can further increase the potential of tumor metastasis. The causal role of PD-L1 in promoting EMT has been confirmed in gastric carcinoma that IFN- γ upregulates the expression of PD-L1 in solid tumor cells through JAK signal transducer and transcriptional pathway activator, and weakens the cytotoxicity of tumor antigen specific CTL to tumor cells. After pretreatment with IFN- γ, cells were treated with anti-PD-L1 mAb, the decreased anti-tumor CTL activity by IFN- γ reached a level higher than that of non-therapeutic control target (45). Mir-940/Cbl-b/STAT5A axis up regulates the expression of PD-L1 and promotes the proliferation and EMT of GC cells (63). In another study, it is also confirmed that EMT can enhance the migration and invasion of GC cells through nuclear factor κB (NF-κB) pathway upregulates PD-L1 (64). Another study showed that the induction of EMT and PD-L1 expression was bidirectional. The induction of EMT upregulates the expression of PD-L1 mainly by activating PI3K-Akt pathway. These results demonstrate that the high expression level of PD-L1 is directly related to the phenotype of epithelial mesenchymal transformation.

### 3.2 PD-L1 and GC stem cell potential

The high expression of PD-L1 in GC can increase the potential of gastric CSCs and further promote the proliferation and progression of GC. Gastric CSCs also express PD-L1 (65). It is reported that GC cells were heterogeneous, and that PD-L1 in GC cells had different reactivity to GCMSCs and PD-L1 associated with CTCF to contribute to the stemness and self-renewal of GC cells, and GC mesenchymal cells (GCMSCS) enhance the CSC like characteristics of GC cells through PD-L1, resulting in the resistance of GC cells to chemotherapy (62). *In vivo*, PD-L1 positive GC cells have greater stem cell potential and tumorigenicity than PD-L1 negative GC cells (62). We find that the specific mechanism between PD-L1 and CSC in tumor cells in the latest study this year, that, the interaction between PD-L1 and frizled 6 receptor up-regulates β-Catenin targeted gene expression and promotes cancer progression through the maintenance and expansion of CSC (66).

### 3.3 PD-L1 and Apoptosis/Autophagy

The most famous biological function of PD-1/PD-L1 axis is to promote apoptosis of effector T cells, the basic principle behind which, is tumor immune escape. In 1992, Ishida et al. found PD-1 expressed transiently during inducing T cell death apoptosis in mice (67). In 2002, Minato et al. found that PD-L1 and PD-1 signaling pathways are involved in the regulation of tumor immunity for the first time (68). It’s reported that endogenous PD-L1 expression in gastric adenocarcinoma epithelial cells can promote T cell apoptosis (69). In another study, exogenous PD-L1 from GC can also induce Jurkat T cell apoptosis and inhibit T cell activation (50). PD-L1 can activate epidermal growth factor receptor (EGFR) through mir-429 to resist apoptosis induced by tumor necrosis factor-related apoptosis-inducing ligand (TRAIL) in GC (20). The most classic mechanism of PD-L1 promoting apoptosis and inhibiting T cell activation can be explained as follows: after PD-L1 binding to PD-1, tyrosine residues located in ITSM of PD-1 are phosphorylated and protein tyrosine phosphatases (PTPs), such as SHP2, are recruited. These PTPs can dephosphorylate various key signal kinases and resist the positive signal events caused by the co-stimulation of TCR-CD28 receptor during T cell activation. To some extent, they preferentially inhibit the pathways driven by TCR rather than the CD28 mediated pathway. We also find an important role of PD-L1 in another cell death, autophagy. In general, autophagy is negatively controlled by the PI3K/Akt/mTOR pathway, and PD-L1 induced activation of this pathway seems to lead to the down regulation of autophagy. It is recently reported that autophagy can inhibit the expression of PD-L1 in GC cells reversely (48). Another study has explored the mechanism that autophagy downregulates PD-L1 by reducing the expression of histone deacetylase (HDAC) (70).

### 3.4 PD-L1 expression and Chemotherapeutic drug resistance

A recurring observation among phase III gastric and esophageal trials combining chemotherapy and immunotherapy has been differential activity based on PD-L1 expression (71). Some studies have reported that PD-L1 can reduce the cytotoxicity sensitivity to cytokine induced killer cell (CIK) therapy in GC (72). The expression of PD-L1 can also enhance cisplatin resistance in GC (73). Low dose Diosbulbin-B (DB) (12.5 μ M) activates tumor-intrinsic PD-L1/NLRP3 signaling pathway to restore the sensitivity of the cisplatin resistant GC (CR-GC) cells to cisplatin, the level of PD-L1 downregulates simultaneously in GC cells (74). These results further confirm that PD-L1 can reduce the sensitivity of GC cells to cisplatin. However, one research reports that non-responders to XELOX chemotherapy, all of whom were PD-L1 negative, failed to upregulate PD-L1 expression on treatment or favorably remodel their TME and exhibited minimal change in T-cell infiltration but rather an increase in LAG3 expression and B-cell infiltration (75). The relationship between PD-L1 expression and cytotoxic chemotherapy response has not been examined in detail, but response is numerically lower (41% vs. 46%) in PD-L1–negative patients in the large (n = 1,581) phase III CheckMate-649 trial (76).

## 4 Clinical application of PD-L1/PD-1 inhibitors in GC

Systemic chemotherapy, radiotherapy, surgery, immunotherapy, and targeted therapy all have proven efficacy in gastric adenocarcinoma; therefore, multidisciplinary treatment is paramount to treatment selection. Triplet chemotherapy for resectable gastric cancer is now accepted and could represent a plateau of standard cytotoxic chemotherapy for localized disease (77). Classification of gastric cancer based on molecular subtypes is providing an opportunity for personalized therapy. The expression level of PD-L1 in GC patients with different molecular types differs. According to the PD-L1 score in gastric cancer, anti PD-L1/PD-1 inhibitor drugs are recommended respectively. The use of pembrolizumab as a third line treatment for patients with PD-L1 positive (CPS ≥ 1) gastric adenocarcinoma is based on many important clinical trials (77), which are described in **Table 1**. According to the Cancer Genome Atlas Project (TCGA), EBV positive tumors were not considered as a separate entity but they largely fell into the MSS/TP53 + and MSS/EMT subgroups. While they differ in terms of etiopathogenesis, molecular characteristics and determinants of immune-sensitivity, the MSI and the EBV subtypes (or EBV positive tumors in the ACGR classification) have been recognized as the gastric cancer subtypes that could benefit most from ICIs (78–80).

**Table 1:** Clinical trials of PD-L1/PD-1 inhibitors in GC.

Trial	Phase (advanced)	Drugs	Actual enrollment	Study period
1^st^ line				
NCT02494583 (KEYNOTE-062)	III; G/GEJ	Pembrolizumab vs Pembrolizumab + Chemotherapy vs Chemotherapy	763	July 31, 2015 - June 6, 2022
NCT03675737 (KEYNOTE-859)	III; G/GEJ	Pembrolizumab+ Chemotherapy vs Placebo + Chemotherapy	1579	8 November 2018 - 28 September 2024
NCT04082364 (MAHOGANY)	II/III; HER2+ G/GEJ	Combination Margetuximab, Retifanlimab, Tebotelimab, and Chemotherapy	81	September 30, 2019 - December 2023
NCT03615326 (KEYNOTE-811)	III; HER2+ G/GEJ	Pembrolizumab/Trastuzumab/Chemotherapy vs Trastuzumab/Chemotherapy	732	October 5, 2018 - March 20, 2024
NCT02901301	Ib/II; HER2+	Pembrolizumab + Trastuzumab + Capecitabine + Cisplatin	41	February 6, 2017 - October 2021
NCT02954536	II; HER2+ Metastatic EGC	Pembrolizumab+trastuzumab + chemotherapy	37	November 3, 2016 - November 2022
ATTRCTION-04	II/III; HER2-; G/GEJ	SOX/Capecitabine plus Oxaliplatin with vs without Nivolumab	724	March 7, 2017 - May 10, 2018
NCT03745170	III; G/GEJ	Sintilimab + XELOX vs XELOX	650	December 19, 2018 - August 31, 2022
NCT02872116 (CHECKMATE-649)	III; G/GEJ	Nivolumab Plus Ipilimumab or Nivolumab in Combination With Oxaliplatin Plus Fluoropyrimidine vs Oxaliplatin Plus Fluoropyrimidine	2031	May 27, 2020- May 31, 2024
NCT03777657	III; G/GEJ	Tislelizumab + Chemotherapy vs Placebo + Chemotherapy	997	December 13, 2018 - August 2022
NCT03472365	II	SHR-1210 in Combination With Capecitabine + Oxaliplatin or Apatinib	67	April 2, 2018 - November 25, 2020
NCT02915432	Ib/II; Advanced GC, ESCC, NPC, HNSCC	Toripalimab (JS001) vs Toripalimab + CapeOx	401	December 2016 - April 30, 2022
NCT02937116	Ib; Advanced Solid Tumors	Sintilimab + CapeOx	233	October 19, 2016 - January 2022
NCT03469557	II	Tislelizumab + Chemotherapy	30	July 18, 2017 - August 19, 2020
NCT03852251	Ib/II; G/GEJ	AK104(a PD-1/CTLA-4 Bispecific Antibody) + XELOX	112	January 18, 2019 - June 2021
NCT02625610	III; G/GEJ	Avelumab vs Oxaliplatin + 5FU/LV or Oxaliplatin + Capecitabine	499	December 24, 2015 - June 3, 2021
NCT01585987	II; G/GEJ	Ipilimumab or Best Supportive care	143	July 2012 - April 2015
2^nd^ line				
NCT03609359 (EPOC1706)	II	Lenvatinib + Pembrolizumab	29	October 15, 2018 - April 1, 2021
NCT03959293 (CheckMate-032)	I/II	Nivolumab vs Nivolumab+ipilimumab vs	107	July 17, 2019 - July 31, 2024
NCT02370498 (KEYNOTE-061)	III; G/GEJ	Pembrolizumab vs Paclitaxel, PD-L1 (+)	592	May 11, 2015 - June 10, 2021
NCT03019588 (KEYNOTE-063)	III; G/GEJ	Pembrolizumab vs Paclitaxel	94	February 16, 2017 - June 29, 2021
NCT02999295	I/II; G/GEJ	Nivolumab + Paclitaxel + Ramucirumab	46	January 2017- August 2019
NCT02335411 (KEYNOTE-059)	II	Pembrolizumab	318	February 3, 2015 - July 23, 2021
NCT02942329	I	Camrelizumab + Apatinib	60	October 2016 - October 2018
3^rd^ line				
NCT01848834 (KEYNOTE-012)	Ib	Pembrolizumab	297	May 7, 2013 - June 30, 2020
NCT02335411 (KEYNOTE-059)	II	Pembrolizumab	318	February 3, 2015 - July 23, 2021
NCT01928394 (ATTRACTION-2)	III	Nivolumab vs placebo	493	November 4, 2014 - February 26, 2016
Late line				
NCT02340975	Ib/II; G/GEJ	Durvalumab + Tremelimumab	114	March 31, 2015 - April 29, 2019
NCT03406871	Ib; GC/CRC	Regorafenib + Nivolumab	50	January 25, 2018 - November 26, 2020
NCT03797326	II; solid tumor	Lenvatinib + Pembrolizumab	590	February 12, 2019 - December 22, 2023
NCT03221426 (KEYNOTE-585)	III; G/GEJ	perioperative Chemotherapy (XP/FPFLOT) with or without Pembrolizumab	1007	October 9, 2017 - June 28, 2024
NCT02743494 (CheckMate-577)	III; GEJ	Nivolumab vs Placebo	794	July 14, 2016 - October 11, 2025

GC, gastric cancer; G/GEJ, gastric cancer/gastroesophageal junction; HER2+, human epidermal growth factor positive; EGC, Esophagogastric cancer; SOX, S-1+oxaliplatin; XELOX, Capecitabine and Oxaliplatin; FOLFOX, 5-fluorouracil/leucovorin plus oxaliplatin; ESCC, Esophageal squamous cell carcinoma; NPC, nasopharyngeal carcinoma; HNSCC, head and neck squamous cell carcinoma.

### 4.1 Clinical trial of PD-L1/PD-1 inhibitors in patients with advanced GC

In different clinical trials of PD-1/PD-L1 inhibitors, most clinical trials have benefited from OS and PFS to varying degrees. ATTRACTION-4 and CHECKMATE-649 trials suggest that the combination of nivolumab with chemotherapy might be more effective than chemotherapy alone (76, 81). KEYNOTE-062, a phase 3 randomized clinical trial demonstrated that pembrolizumab was noninferior to chemotherapy for OS in patients with CPS of 1 or greater, but pembrolizumab monotherapy was not superior to chemotherapy in patients with CPS of 1 or greater (82). However, there have also been certain cases of failure. For example, the use of avelumab alone in the third-line treatment of GC patients can not significantly improve the overall survival (OS) of patients compared with the chemotherapy drugs selected by doctors. However, the results of Javelin gastric 300 (clinical trial NCT02625610) provide an important reference for us to continue to explore the role of avelumab in the treatment of GC. According to different drug regimens of PD-L1/PD-1 inhibitors, we divided them into first-line, second-line, third-line, and late treatment according to the combination of Europe, the USA, and Asia guideline for gastric cancer, as shown in **Table 1**.

### 4.2 Clinical therapy of PD-L1/PD-1 inhibitors for different molecular types of GC

**Figure 3:**
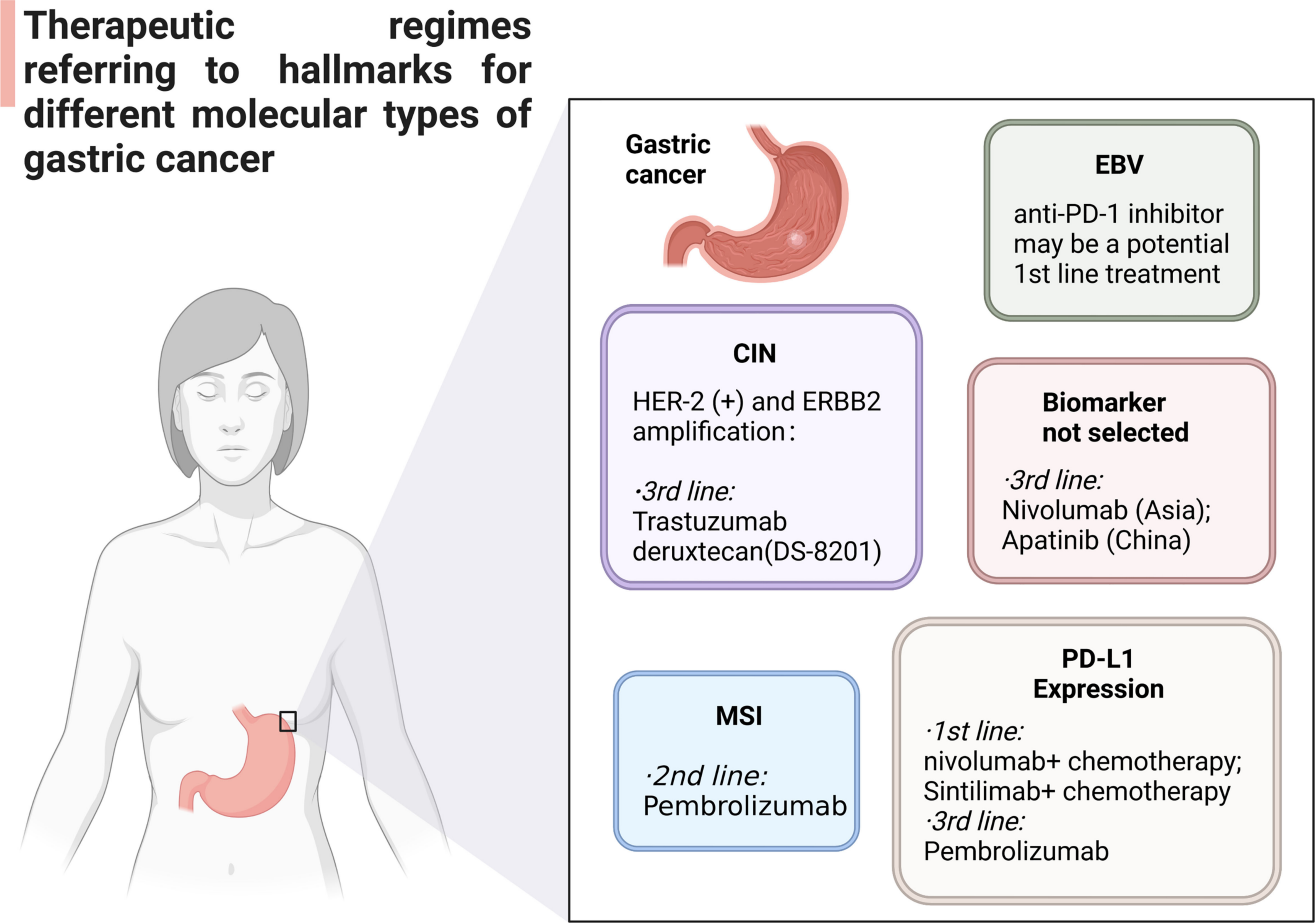
Anti-PD-L1/PD-1 regimens in different molecular typing of GC patients.

Chromosomal events in patients with CIN GC include HER2 + or ERBB2 amplification, FGFR2b expression, FGFR amplification, EGFR amplification and MET amplification. Trastuzumab plus chemotherapy is the first-line drug for GC patients with HER-2 (+) and ERBB2 amplification in the current medical guidelines (**Figure 3**). Trastuzumab Deruxtecan DS-8201 (China) has been reported as a third-line drug for HER2 + GC (83). ADC targeting HER2 and immune checkpoint inhibitor are important potential biomarkers for clinical treatment, as well. Pembrolizumab currently be used in the second-line treatment of MSI GC. Tumor tissues were collected from patients participating in ITACA-S, and randomized adjuvant chemotherapy trials were carried out. According to MSI status, inflammatory response, and the expression of PD-L1, the prognosis and regressive analysis of MSI GC were analyzed. The results show that MSI status may be a meaningful prognostic biomarker in patients with radical resection stage II-III GC. The first-line regimens of PD-1/PD-L1 inhibitors currently used in GC patients expressing PD-L1 are: (1) nivolumab plus chemotherapy (CPS ≥ 5%); (2) Sintilimab plus chemotherapy (China). Third-line therapeutic schedule is pembrolizumab (CPS ≥ 1%). PD-1 monoclonal antibody may also be used as a potential first-line regimen for the treatment of MSS/TMB-high GC. At present, there is no suitable PD-1/PD-L1 agents for EBV positive GC, but PD-1 monoclonal antibody may be a potential first-line treatment for EBV cytotoxic T lymphocytes. Nivolumab (Asia) and Apatinib (China) can be used as a third-line regimen for some GC patients without clear molecular classification. At the same time, PD-1 monoclonal antibody combined with multiple kinase TKI (Stivarga/Lenvatinib) and PD-1 monoclonal antibody combined with PARP inhibitor can also be used as candidate immunotherapy schemes for this kind of groups.

### 4.3 Application of PD-L1/PD-1 inhibitor combined with other therapies in GC

As many of the patients with GC are PD-L1 negative, combination therapy is attractive. Furthermore, even some patients who are PD-L1 positive have quite limited benefit from anti-PD-1 therapy alone and relapse/recurrence frequently occurs due to various resistance mechanisms (84). The limitation of anti PD-L1/PD-1 monotherapy is due to the complex tumor microenvironment (TME) and multiple immunosuppressive factors in each step of the cancer immune cycle (85). In order to achieve effective tumor control and eradication, three levels of immunotherapy capabilities are required. First, CD4+T, CD8+T and B lymphocytes are activated to become antigen specific effector cells. Second, the inhibitory factors in the turbine microenvironment, represented by PD-L1 and others, are neutralized. Third, tumor cells that express related targets are recognized and killed (86). Therefore, increasing the benefit of checkpoint-immune blockade through combinations to achieve long-term efficacy may be helpful (87, 88). At present, PD-1/PD-L1 inhibitors combined with various chemotherapeutic drugs or targeted regimens have been reported in clinical trials.

#### 4.3.1 Combined with traditional chemotherapeutic drugs

Purpose of current immunotherapy is to change the immunogenicity of tumor cells, turn “cold” tumors into “hot” tumors, make more prominent T cells and cytotoxic T cells infiltrate around the tumors, reduce the density and number of corresponding immunosuppressive cells, and further increase the density of TILs and the concentration of chemokines in the tumor microenvironment. The results of such treatment strategies have also been well repeated under the treatment of many chemotherapeutic drugs (89). Some studies have shown that the potential mechanism of the synergistic effect of chemotherapeutic drugs and immune checkpoint inhibitors may include the following: (1) induced immunogenic tumor cell death, (2) anti-angiogenesis, (3) selective depletion of myeloid immunosuppressive cells, (4) loss of lymphopenia (90). The commonly used chemotherapy regimens for GC include Cisplatin, Oxaliplatin, Adriamycin, 5-Fluorouracil, Paclitaxel and Docetaxel. In-ho Kim et al. reported that PD-L1 positive GC patients owned down-regulated PD-L1 expression level after treated with cisplatin and had a better clinical prognosis (91). 5-FU, the most widely used chemotherapy drug for GC, can increase the expression of PD-L1 in GC cells and reduce bone marrow mesenchymal stem cells, and then weaken the CSC dryness of tumor cell. The latest clinical trial (NCT04065282) results show that as a neoadjuvant chemotherapy regimen, Sintilimab combined with oxaliplatin and capecitabine can significantly improve the postoperative remission rate of patients with advanced resettable GC (92).

However, not all chemotherapy plus immunotherapy will bring better prognosis to patients with GC. A randomized phase III trial demonstrated that patients with GC expressing PD-L1 had a worse prognosis after pembrolizumab combined chemotherapy, which includes cisplatin, fluorouracil and capecitabine (82). Therefore, specific problems need to be analyzed to determine the appropriate synergistic treatment through accurate clinical trials.

#### 4.3.2 Combined with radiotherapy

Radiotherapy (RT) is used to eliminate tumor cells by ionizing radiation site-specifically. The immune principle of radiotherapy for tumors also includes that radiation can induce immunogenic cell death (ICD), so that tumor antigen can be presented and promote T cells to kill tumor cells (93). However, radiotherapy does not always have an immune promoting effect on tumors. Due to immunosuppressive cytokines, like TGF- β and IL-10, and chemokines, in the process of radiation, tumor cells can also interact with immunosuppressive related cells (94). Here, surprisingly, we found that radiotherapy also had a certain correlation with PD-L1 expression. It has been revealed that after gastrointestinal cancer cells receive fractionated radiotherapy, T cells, referring to CD8 (+) T cells mostly, in the tumor microenvironment, have capacity to secrete IFN-γ to induce the up-regulation of PD-L1 in tumor cells (95). In these reports, most tumor patients with up-regulated PD-L1 after radiotherapy commonly have a worse clinical prognosis. As for the combination of radiotherapy and immunotherapy, in non-small cell lung cancer, the combination of RT and PD-1 inhibitor can significantly inhibit tumor growth and prolong survival (96). These results leave us enlightenment whether it is necessary to combine radiotherapy and PD-1/PD-L1 inhibitors in GC, and whether it may benefit patients.

#### 4.3.3 Combined with targeted therapy

GC is a typical heterogeneous tumor, whose high expression of HER2+ own similar targeting regimen scheme compared with HER2+ breast cancer. It has been reported that the combination of PD-1 inhibitor and anti-HER2 treatment can induce T cell activation and promote tumor inhibition (97). In several clinical trials of PD-1/PD-L1 inhibitors against HER2+ GC mentioned above, keynote-811, NCT02901301 and NCT02954536 have revealed a clinical result that such combinations can show a certain advantage in inhibiting progress of solid tumors. Currently, the combination of PD-1/PD-L1 inhibitors and small molecule inhibitors has been reported in many literatures and clinical trials, which can bring great benefits to different kinds of cancer patients, including nivolumab, pembrolizumab, PDR001, TSR-042, avelumab, atezolizumab, etc. Although no specific combination of PD-1/PD-L1 immune checkpoint inhibitors and small molecule inhibitors has been reported in GC, we still can learn the relevant details of drug combination in different malignant diseases in other studies (98). Among them, small molecule inhibitors used in combination with anti-PD-1/PD-L1 agents include but are not limited to EGFR inhibitors, vascular endothelial growth factor(VEGF/VEGFR) inhibitors, PI3K and MAPK pathway inhibitors, DNA methyltransferase (DNMT) inhibitors and histone deacetylase (HDAC) inhibitors, indolamine 2,3-dioxygenase 1(IDO1) inhibitors poly ADP ribose polymerase (PARP) inhibitor, Bruton’s tyrosine kinase inhibitor, TGF β Receptor I (TGF-β RI), MDM2 inhibitor, CHK1 inhibitor, etc. Such kind of combination ought to be one sort of great research trend in the future, because the basic research on the relationship between these targets and PD-L1 is in full swing. In addition to the combination of immunotherapy, there are reports on the novel treatment of nano new drugs carrying PD-L1 monoclonal antibody. Cell-uptake experiment further confirmed that NPs combined with PD-L1 monoclonal antibody can improve the uptake of the drug, suggesting the possibility of improving the drug regimen for clinical GC patients (99).

#### 4.3.4 Dual immune checkpoint inhibitor (ICI) strategy

Immunosuppressive molecules like cytotoxic T lymphocyte antigen 4 (CTLA-4) and lymphocyte activation gene 3 (LAG3) are also popular and widely used besides PD-1. In GC, the combination of anti-PD-1 and anti-CTLA4 has been tested in clinical trial that checkmate-032 clinical trial revealed great clinical result of this combination (100). Meanwhile, the combination of anti-PD1 and anti-CTLA4 can significantly inhibit the proliferation, apoptosis, migration, invasion and EMT in MKN45 GC cells (101). Clinically established combination therapies have been approved for the treatment of metastatic melanoma, advanced renal cell carcinoma and metastatic colorectal cancer with MMR/MSI-H distortion, such as CTLA-4 inhibitor ipilimumab and PD-1/PD-L1 inhibitor nivolumab.

The synergy between LAG3 and PD-1 is still in clinical trials, although there is currently no such regimen as a clinical guideline for frontline medication for GC patients. However, the effectiveness and possibility of such combination therapy have been reported in the previous documents. In ovarian cancer (102), lymphoma (102) and melanoma (103), combination of anti-LAG3 and anti-PD1 can lead to significant tumor regression.

Aiming at PD-L1 expressed in tumor cell surface and PD-1 targets expressed in T cells of immune system, Innovent Biologics, Inc. has launched bis-specific combined drug, made solid fundamental research in terms of its pharmacokinetics and metabolomics, and is currently carrying out clinical trials (104). However, the disadvantages of this bispecific antibodies with anti-PD-1 or anti-PD-L1 arms may exist: (1) inaccurate drug targeting and easy to produce off target effect (2) the drug may have the same ADCC toxic effect on T cells induced by natural kill (NK) cells as tumor cells.

### 4.4 Progress, side effects and drug resistance of PD-L1/PD-1 inhibitors in GC

In terms of mentioned above dual ICIs treatment scheme, the application of immune checkpoint inhibitors can amplify the immune response, T lymphocytes were over-activated, leading to the immune-related adverse events (irAEs). Gastrointestinal tract, lung, joint and other organs could be injured, such as inflammatory arthritis, synovitis, synovitis and Sjogren’s syndrome, antinuclear antibody positive and other immune diseases would appear. Patients treated with anti-PD-1/PD-L1 were at greater risk of hypothyroidism, hepatotoxicity, and pneumonia.

The current application of PD-L1/PD-1 inhibitors in patients with advanced GC has greatly improved the prognosis and quality of life of patients (105). Moreover, we found that about 12.5% of the patients who received immunotherapy could benefit which based on the data of patients who met the qualification criteria of ICI treatment in 2018, The proportion of patients who benefit from this is less in GC. Therefore, the drug regimen of PD-L1/PD-1 inhibitor is recommended only for some patients with advanced GC with high expression of PD-L1. In these patients, acquired drug resistance also occurs in the later stage of treatment due to different reasons such as tumor heterogeneity, epitope change, PD-L1 blocking and so on (106).

In addition, the use of ICIs and the benefits of patients may also be related to the intestinal microbial environment. Recently, it has been reported that intestinal flora may affect the therapeutic effect of anti PD-1 inhibitors on patients through the level of tumor microenvironment (107), which has been further verified in patients with melanoma (108). Intestinal microbiome analysis showed a significant increase in bifidobacterial species in mice with slow tumor growth and the consequent increased benefit of PD-1 therapy (107, 109). The intestinal diversity of patients who responded to PD-1 treatment significantly increased their microbiota, some of which were relatively rich, such as Clostridium and Fecal bacilli (108). At present, it is not clear which specific composition of intestinal microbiota is most conducive to promoting anti-tumor immune response or promoting immunotherapy where the research on intestinal microbial ecology and tumor microenvironment is in full swing.

Immunotherapy such as anti-PD-1/PD-L1 further leads to the rapid progression of some tumors, which is called hyper progressive disease (HPD). The incidence rate of HPD in AGC is not clear, but the risk is certain. It was previously reported that when nivolumab was administered to AGC patients, liver metastasis and size of tumor at baseline were significantly associated with HPD. Some researchers explained that it may be necessary to further replace the concept of HPD with super HPD (naturally rapid growth of tumor after treatment) (110). At the same time, a separate case reported the occurrence of HPD in patients with advanced GC treated with nivolumab (111). However, it has been reported that there is no statistical correlation between PD-L1 expression in GC and hyper-progressive diseases (112).

In addition to the corresponding side effects, it has also been reported that new nanoparticles composed of platinum loaded complexes can produce positive synergistic effects in a large dose range, thereby achieving higher immunotherapeutic effects and lower side effects (113). MSI-H/dMMR phenotype, high tumor mutational burden and EBV etiopathogenesis appear to be more reliable indicators of tumor immunogenicity and sensitivity to PD-1L/PD-1 inhibitors. Indeed, PD-L1/PD-1 inhibition can substantially improve the outcome of these kinds of GC patients, the problem to be solved is to better identify the patients and the corresponding inclusion criteria for PD-L1/PD-1 therapy, while further clinical trials are needed to explore such specific biomarkers (114).

## 5 Conclusions

The immunogenicity of gastric cancer varies through molecular types. We classified it by different molecular types and corresponding clinical trial results, and listed several clinical trials of PD-L1 inhibitors and corresponding prognosis results. We believe that in the current clinical guidelines, patients with relatively high immunogenicity of gastric cancer types can be treated with PD-L1 inhibitors to achieve better efficacy under the premise of excluding some cases of acute side effects. With the exploration of various basic articles, the regulatory mechanism of PD-L1 cells has gradually been recognized more clearly. We provide the latest mechanistic regulatory pathway, which is described as PD-L1 can be regulated by various star signals inside and outside the cell, including IFN-r and some miRNAs. These regulation pathways are reflected in transcriptional, translational, and epigenetic levels, which provides a good thinking for the future development of clinical drugs.

First choice for operable GC patients should be surgery. At present, for patients with advanced GC, our treatment guidelines have clearly adopted the use of ICIs. In the case of combined chemotherapy and other drugs, obvious benefits have been brought to patients with AGC. On this basis, anti-PD-L1/anti-PD-1 treatment for patients with GC has been significantly beneficial in combination, and its application scope is not limited in the phase III guidelines mentioned above. There are many aspects to be considered such as irAEs and super progression events in the use of anti-PD-L1/anti-PD-1 regimes as systemic immunotherapy for GC patients, but it still has been highly valued, which may improve the survival time and quality of life of patients to varying degrees.

## Author contributions

YZ contributed to the Conceptualization, Writing – original draft. YY, YC, WL, XC, JL, and YH contributed to the Writing – review & editing. HW and LT contributed to the Supervision, Validation, Writing – review & editing. All authors contributed to the article and approved the submitted version.

## Funding

This work was supported by Science and Technology Program of Zhejiang Province [2021c03119] and Natural Science Foundation of Zhejiang Province [LQ18H160012].

## Acknowledgments

Figures created with BioRender.com.

## Conflict of interest

The authors declare that the research was conducted in the absence of any commercial or financial relationships that could be construed as a potential conflict of interest.

## Publisher’s note

All claims expressed in this article are solely those of the authors and do not necessarily represent those of their affiliated organizations, or those of the publisher, the editors and the reviewers. Any product that may be evaluated in this article, or claim that may be made by its manufacturer, is not guaranteed or endorsed by the publisher.

## Glossary

**Table:**

5-FU	5-fluorouracil
ACT	adoptive cell immunotherapy;
AGC	advanced GC
ATRA	all trans retinoic acid
CAPOX/XELOX/CapeOx	capecitabine and oxaliplatin
CIK	cytokine induced killer cell
CIN	chromosomal instability
CPS	comprehensive positive score
CR-GC	cisplatin resistant GC;
CSC	cancer stem cell
CSF-2	colony stimulating factor 2
CSN5	COP9 signalosome 5
CTLA-4	cytotoxic T lymphocyte antigen 4
DB	Diosbulbin-B
DFS	disease-free survival
DNMT	DNA methyltransferase
EBV+	Epstein-Barr virus positive
EGFR	epidermal growth factor receptor
EMT	epithelial mesenchymal transformation phenotype
FOLFOX 5- fluorouracil	leucovorin and oxaliplatin
GC	Gastric cancer
GCMSCS	Gastric cancer mesenchymal cells
GEA	gastroesophageal adenocarcinoma
GEJ	gastroesophageal junction
GM-CSF	granulocyte-macrophage colonystimulating factor
HDAC	histone deacetylase
HER2	human epidermal growth factor
HNSCC	head and neck squamous cell carcinoma
HPD	hyper progressive disease
HPV	human papillomavirus vaccine
ICD	immunogenic cell death
ICI	immune checkpoint inhibitor
IDO	indoleamine dioxygenase;
IFN	Interferon
IL	Interleukin
JAK	Janus kinase
LAG3	lymphocyte activation gene 3
LMP1	latent membrane protein 1
MDSCs	myeloid-Derived Suppressor Cells
MM	multiple myeloma
MSI	Microsatellite instability
NF- kB	nuclear factor kB
NK	natural kill
Nps	nanoparticles
NSCLC	non-small cell lung cancer
OA	oleanolic acid
OS	overall survival
PARP	poly ADP ribose polymerase
PBMCs	Peripheral blood mononuclear cells
PBML	peripheral blood mononuclear lymphocyte
PD-1	programmed death-1
PD-L1	programmed cell death-Ligand 1;
PD-L1	Programmed death-ligand 1
PFS	progression freesurvival
PTPs	protein tyrosine phosphatases
RFS	relapse free survival
RT	radiotherapy
SREBP	sterol regulatory element binding protein
STAT3	signal transducer and activator of transcription 3
TAAs	tumor associated antigens
TGF-b	transforming growth factor b
TIL	tumor infiltrating lymphocytes
TLR-3	toll like receptor 3
TNF-a	tumor necrosis factor a
TPS	tumor proportion score
TRAIL	tumor necrosis factor related apoptosis-inducing ligand
TTP	Tristetraprolin
USP7	ubiquitin specific peptidase
VEGF	vascular endothelial growth factor
